# Comprehensive Imaging of Sensory-Evoked Activity of Entire Neurons Within the Awake Developing Brain Using Ultrafast AOD-Based Random-Access Two-Photon Microscopy

**DOI:** 10.3389/fncir.2020.00033

**Published:** 2020-06-16

**Authors:** Kelly D. R. Sakaki, Kaspar Podgorski, Tristan A. Dellazizzo Toth, Patrick Coleman, Kurt Haas

**Affiliations:** ^1^Department of Cellular and Physiological Sciences, Djavad Mowafaghian Centre for Brain Health, University of British Columbia, Vancouver, BC, Canada; ^2^Janelia Research, Ashburn, VA, United States

**Keywords:** comprehensive imaging, random-access, multi-photon, acousto-optics, *in-vivo* calcium imaging, encoding, dendritic integration

## Abstract

Determining how neurons transform synaptic input and encode information in action potential (AP) firing output is required for understanding dendritic integration, neural transforms and encoding. Limitations in the speed of imaging 3D volumes of brain encompassing complex dendritic arbors *in vivo* using conventional galvanometer mirror-based laser-scanning microscopy has hampered fully capturing fluorescent sensors of activity throughout an individual neuron's entire complement of synaptic inputs and somatic APs. To address this problem, we have developed a two-photon microscope that achieves high-speed scanning by employing inertia-free acousto-optic deflectors (AODs) for laser beam positioning, enabling random-access sampling of hundreds to thousands of points-of-interest restricted to a predetermined neuronal structure, avoiding wasted scanning of surrounding extracellular tissue. This system is capable of comprehensive imaging of the activity of single neurons within the intact and awake vertebrate brain. Here, we demonstrate imaging of tectal neurons within the brains of albino *Xenopus laevis* tadpoles labeled using single-cell electroporation for expression of a red space-filling fluorophore to determine dendritic arbor morphology, and either the calcium sensor jGCaMP7s or the glutamate sensor iGluSnFR as indicators of neural activity. Using discrete, point-of-interest scanning we achieve sampling rates of 3 Hz for saturation sampling of entire arbors at 2 μm resolution, 6 Hz for sequentially sampling 3 volumes encompassing the dendritic arbor and soma, and 200–250 Hz for scanning individual planes through the dendritic arbor. This system allows investigations of sensory-evoked information input-output relationships of neurons within the intact and awake brain.

## Introduction

Neurons within brain circuits receive inputs from hundreds to thousands of upstream neurons at synaptic contacts distributed across their elaborate dendritic arbors. Information received in synaptic currents is integrated within dendrites and transformed into neural output encoded in action potential (AP) firing at the soma, which is then transmitted via the axon to downstream targets. Information carried by neural activity can be deciphered by tracking synaptic events or APs evoked by controlled sensory stimuli in awake animals. However, our understanding of neural information processing and encoding is limited due to technical challenges in simultaneously tracking activity throughout complex three dimensional (3D) dendritic arbor structures and the soma, which is needed to establish full input-output relationships.

Approaches to this problem have been aided by the development of fluorescent sensors of neural activity, particularly those capable of detecting rapid changes in intracellular free calcium. Neurons tightly control the transmembrane calcium gradient with intracellular concentrations being on the order of 10,000 times lower than extracellular levels (Gleichmann and Mattson, 2013). Synaptic and AP activity evoke distinct spatiotemporal elevations in intracellular calcium (reviewed in Sabatini et al., 2002; Redmond and Ghosh, 2005). Excitatory, glutamatergic synaptic transmission induces calcium influx by activation of calcium-permeable N-Methyl-D-aspartate (NMDA) and GluA2 subunit-lacking α-amino-3-hydroxy-5-methyl-4-isoxazolepropionic acid (AMPA) subtypes of glutamate receptors (Burnashev et al., 1992), and secondary opening of voltage-gated calcium channels (Sabatini and Svoboda, 2000; Cowan et al., 2001; Oertner and Svoboda, 2002; Sabatini et al., 2002). Transients within the soma from voltage-gated calcium channels are also triggered by AP-mediated depolarization (Nakai et al., 2001; Chen et al., 2013; Gleichmann and Mattson, 2013). Thus, calcium transients can be imaged as a proxy for neural activity using calcium-sensitive fluorescent dyes or genetically-encoded calcium indicators (GECIs) (Nakai et al., 2001; Rose et al., 2014), such as the family of engineered GCaMPs (Chen et al., 2013). These indicators inherently slow neural activity signals due to their calcium binding kinetics (Nakai et al., 2001; Chen et al., 2013), but require sampling rates on the order of milliseconds to adequately measure transient rise times and signal intensity (Katona et al., 2011), and seconds for decay times (Chen et al., 2013; Sun et al., 2013). An alternative strategy for detecting synaptic input has been the development of sensors of neurotransmitters. For sensing glutamate, the genetically encoded protein iGluSnFR can be expressed on the surface of neurons and increases fluorescence upon binding to pre-synaptically-released glutamate (Marvin et al., 2013, 2018).

In order to fully capture a neuron's synaptic and AP neural activity within the intact brain one must image the complex 3D dendritic arbor morphology and soma relatively deep within light-scattering brain tissue at sample rates sufficiently fast to track activity. Conventional two-photon laser scanning microscopy (TPLSM) systems achieve deep imaging up to 600–800 μm into brain through use of long-wavelength excitation light which can penetrate further into the brain than wavelengths used for single-photon absorption in confocal microscopy (Denk et al., 1994; Svoboda et al., 1997). However, the most significant obstacles of conventional TPLSM systems in achieving full imaging of brain neuronal activity is their slow rate of 3D-volume imaging. Inertia-induced latencies are incurred by the galvanometer mirrors used to deflect the laser between imaging points. Conventional systems image brain neurons (e.g., within volumes of 100 μm^3^) using numerous sequences of line-scans (i.e., “rastering”) to image a single 2D X-Y image-plane. Repeating this 2D sampling at multiple incremental steps throughout the Z-axis is employed to fully capture the 3D-volume encompassing the neuron's cell body and dendritic arbor. Although conducting line-scans using galvanometer mirror-based systems is relatively fast between two points of interest (POIs) on one X-Y plane, requiring only single-axis transitions, scanning rates slow dramatically as the number of non-linear POIs increase due to multiple X-, and Y-axis transitions. For example, imaging two points on a 10-μm line on the same plane can achieve scanning rates of 1 attainable reduces to 20 Hz, yet decreases to 1 Hz or slower when the points are spread over an area of 100 μm^2^. Thus, imaging a 3D volume of 100 μm^3^, with 1 μm interval steps per plane on the Z-axis (i.e., 100 X-Y image planes) requires in excess of 100 s per cycle to complete, which is significantly below the rate required for tracking neural activity using calcium biosensors. Critically, the vast majority of sample time conducted by such conventional TPLSM volume imaging of brain neurons is wasted since the structure of neurons comprises a small fraction of the image space. Since neuronal morphology is comprised of long, thin and often highly branched dendrites, the majority of the 3D volume is composed of irrelevant unlabeled extracellular tissue surrounding the target neuron.

Significant recent advances in microscopy have improved the spatial and temporal sampling rates of conventional imaging systems, which have been critical steps toward achieving comprehensive imaging of brain neurons. To increase the ability to change focal depths piezo-actuators have been employed, such as driving the actuator with a sinusoidal voltage, to produce rapid mechanical oscillation of the microscope objective (Göbel et al., 2007). Combined with galvanometer mirror control of the focal point in the X-Y plane, such systems proved capable of capturing somatic calcium transients from a volume containing several hundred cell neurons at 10 Hz. Incremental increases in imaging speeds can be achieved by replacing X-Y rastering with a faster spiral scan pattern, or restricting imaging areas to pre-defined areas containing neuronal elements. Further improvements were achieved by synchronizing the X-Y positioning by the galvanometer mirror with the Z-axis oscillatory motion of a piezo-actuator to scan 3D neuron structures (Katona et al., 2011).

**Figure 1:**
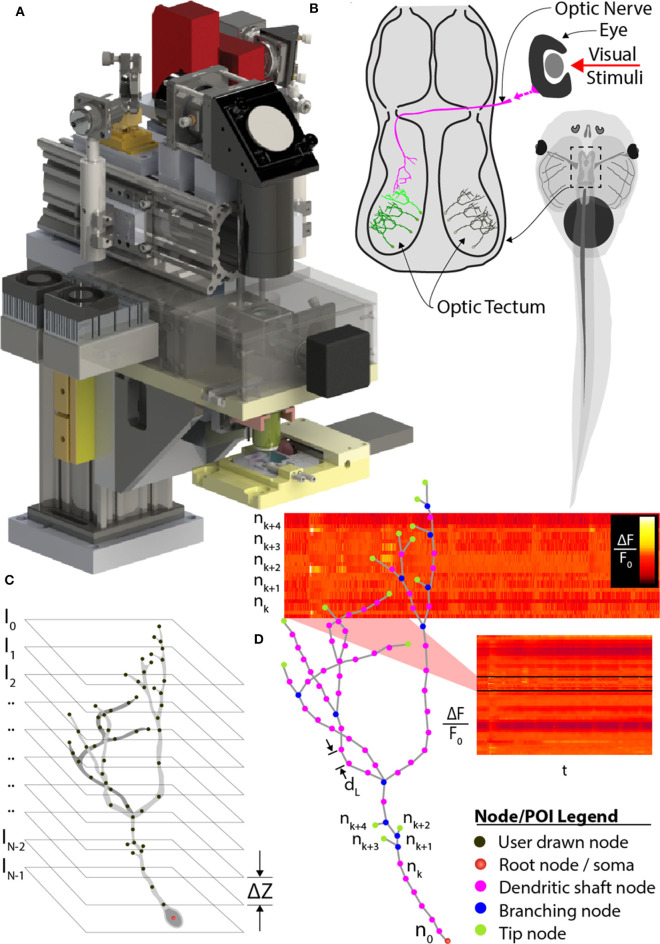
Comprehensive imaging. **(A)** The random-access, two-photon laser-scanning system used to analyze neuron morphology and functional activity. **(B)** Schematic of *Xenopus* tadpole brain visual circuit showing tectal neurons targets (green) for comprehensive imaging *in vivo*. Neurons are labeled with a space-filling fluorophore to capture morphology and a calcium indicator to monitor activity. Neuronal morphology is first determined using a stack of 2D planes encompassing the entire neuron. **(C)** Next, the user traces the 3D dendritic arbor of the neuron, from which points-of-interest (POI) along the entire dendritic arbor and cell body are converted to a 3D tree-structure relationship, and **(D)** automatically interpolated at 2 μm intervals. A “rapid-scan” executes a routine employing AOD-based random-access imaging to simultaneously sample the interpolated POIs to record the AP and all synaptic activity across the neuron.

Significantly increased rates of imaging complex 3D neuronal structures have been achieved using “random-access” sampling, which allows discrete POI sampling, and scanning multiple POIs in 2- or 3D without scanning the intervening space between them (Figure 1). Random access microscopy takes advantage of acousto-optic deflectors (AODs) in place of galvanometer mirrors (Bullen and Saggau, 1997, 1999; Iyer et al., 2005; Salomé et al., 2006; Reddy et al., 2008; Grewe et al., 2010; Katona et al., 2012; Nadella et al., 2016; Szalay et al., 2016). Soundwave-mediated changes in the AOD crystal refractive index acts as a diffraction grating to refract the path of the laser, producing a rapid, inertia-free laser position transition system. Systems using pairs of AODs can achieve kHz-rate sampling in X-Y planes for both one- and two-photon imaging (Bullen and Saggau, 1997, 1999; Iyer et al., 2005; Salomé et al., 2006), and two pairs of AODs can be used for X-Y and Z-axes transitions (Reddy et al., 2008; Katona et al., 2012; Nadella et al., 2016; Szalay et al., 2016). AODs have also been recently applied as laser beam shapers, which could serve systems that require very fast wavefront control (Akemann et al., 2015). AOD-based microscopy has been applied to imaging multiple dendrites and the soma of individual neurons *in vivo* in rodents (Szalay et al., 2016). Fast imaging of cubes or rectangular volumes encompassing the target neuronal compartments was used to compensate for the motion artifacts inherent in mammalian models due to blood flow and respiration. Other approaches incorporate remote focusing to speed Z-axis positioning enabling line-scanning *in vivo* within a volume spanning over two hundred microns (Nadella et al., 2016).

However, application of fast-scanning technologies for comprehensive imaging of fluorescent indicators of sensory-evoked neural activity across an entire neuron *in vivo* remains a challenge. Here, we approached this problem by designing a random-access AOD-based TPLSM system with a variable-speed piezo-actuator system for Z-axis focus, combined with *a priori* determination of the neuronal structure to direct the image path, as well as by selecting a model system with minimal drift and brain neurons with relatively small dendritic arbors. We have selected the albino *Xenopus laevis* tadpole as a model system since they are readily immobilized under awake conditions, and their transparency allows direct visualization of neurons without the brain tissue movement intrinsic to mammals. We fluorescently label tadpole brain neurons using *in vivo* single-cell electroporation to transfect neurons with space-filling fluorophores or biosensors of activity (Haas et al., 2001). Our approach involves first imaging the brain volume encompassing a target neuron using serial sectioning (Figure 1C), and then creating a schematic rending of the soma and dendritic arbor to provide the *a priori* knowledge of the full 3D neuronal structure to direct discrete fast random-access sampling (Figure 1D). This *a priori* structure must be determined for each neuron imaged, since individual brain neurons have unique patterns of dendritic branches extending in non-uniform paths (Haas et al., 2006; Chen et al., 2010; Romand et al., 2011; Hossain et al., 2012). We provide examples for application of our AOD-based random access TPLSM system for imaging of visual-evoked neural activity of individual brain neurons within the optic tectum of awake, immobilized tadpoles. We describe the software driving this microscope and alternative modes of sampling neural activity to optimize coverage or sample rate.

## Methods

### AOD-Based Random-Access Microscope Optical Train

**Figure 2:**
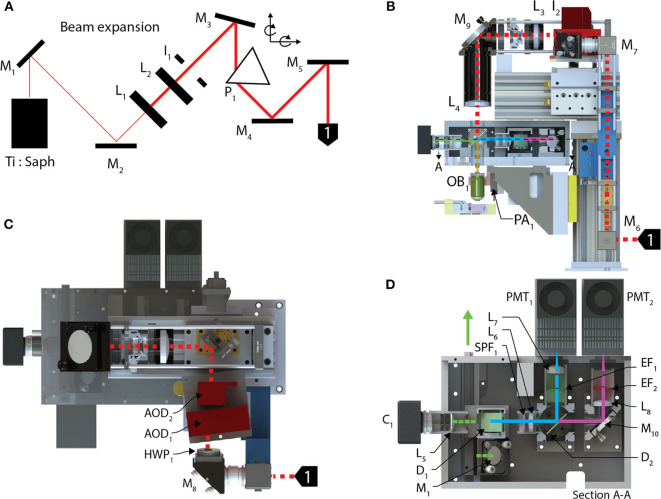
Optical train and the stimulation module. **(A)** The pre-processing optical train for the AOD TPLSM undergoes 4X beam expansion and is passed through a prism used to spatially compensate for the dispersion created in the acousto-optic deflectors deflecting the beam into the back aperture of a 60X objective. **(B)** The beam enters the optical train of the AOD-TPLSM, passes through **(C)** two AODs orientated 90 degrees with respect to each other and provides X-Y scanning. Two lenses collimate the laser at the pupil of the back aperture of the microscope objective. The fluorescence light returns through the objective and **(D)** enters the post-processing optics by reflecting off a dichroic mirror. The emission path on the AOD-TPLSM is split between two channels using a short-pass dichroic mirror. Channel 1, filtered green emissions, and Channel 2 captures red emissions. Calcium transients were observed using jGCaMP7s on the Channel 1. The fluorescence light is detected by a PMT dedicated to each channel.

The optical train (Figure 2) of the random-access system achieves high-throughput POI scanning as well as volume imaging (i.e., multiple X-Y images within a volume region) by using a piezo-actuator, for Z-axis motion, and two AODs to provide *X-, Y*- axis scanning. In combination, this allows for sampling at any possible scan points in three dimensions. In our system, the X and Y axes were scanned using two wide-scan angle AODs, *AOD*_1, 2_ (OAD1121-XY deflector / DA104-2 power driver, Isomet) for NIR with a scan angle of 5.4 degrees, an *T*_*ACC*_ of 13 μs, 40 MHz bandwidth and 9 mm apertures. Transitioning between any two points on the image plane using the AODs executes at rates up to 100 kHz. A Ti:Sapphire laser (Chameleon Vision II, Coherent), with tuned, temporal-dispersion compensation, provides femtosecond pulses and enters the preprocessing optical train through a dichroic mirror *D*_1_ (T660LPXR-UF2, Chroma) and overfilling the back-aperture of a water immersion objective (60X, 1.1 NA, 0.150 kg, LUMPL, Olympus). The objective is mounted on a piezo-actuator (*PA*_1_, QNP-250-250L, 250 μm range, 1 kg maximum payload, Aerotech Inc.) to adjust the focal plane.

The fluorescent light emitted from the sample returns through the objective and exits from the back aperture pupil, reflects off *D*_1_ and is then filtered through a shortpass filter (*SPF*_1_, 700SP-2P, Chroma) to prevent stray laser emissions. The emitted and collimated light is focused using the relay lens *L*_5_, and then separated into green and red wavelengths by a dichroic mirror, *D*_2_ (565LPXR, Chroma). The green channel, to measure the jGCaMP7s signal, is filtered using a bandpass emission filter (*EF*_1_, ET525/50m-2P, Chroma) and red channel is filtered using a bandpass emission filter (*EF*_2_, ET620/60m-2P (Chroma). The emitted light from each channel passes through the relay lenses, *L*_6_ and *L*_7, 8_, to fit through the apertures of the GaAsP photo-multiplier tubes (H7422-40, Hamamatsu). The PMT signals are amplified using low-noise current amplifiers (SR570, Stanford Research Systems). The signal output of the amplifier was acquired using a 12-bit analog input (PCI-6110 DAQ; National Instruments), at a rate of 2.5 MHz.

### Piezo-Actuator and POI-Based Trajectory

**Figure 3:**
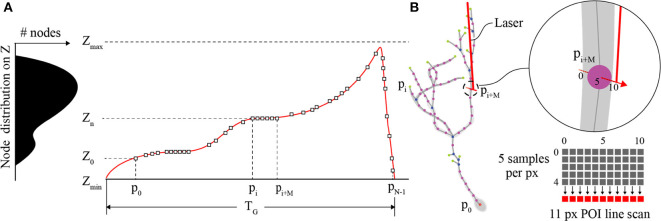
POI distribution and Z-actuator schedule and trajectory. **(A)** A typical distribution of POIs in a tectal neuron and the scanning schedule of POIs synchronizing the activity of the Z-actuator and the AODs. **(B)** An illustration of the neuron structure representing the POIs scanned in A, and an example of three generic POIs on the neuron in 3D space and how they relate to a possible Z-stage trajectory planned for scanning and synchronization with the AODs (inset). The “short” line scan of each POI is created in image space using 11 pixels. The center pixel exists at the location of the POI and each pixel is created from 5 summed samples of the PMT output.

The primary role of the Z-axis actuator for comprehensive imaging is to move the focal plane of the objective to each POI between [Z_min_, Z_max_] on the interpolated neuron in 3D space (Figure 3A). Piezo-actuators are capable of large accelerations with heavy loads over relatively large distances and can provide sub-micrometer repeatability. For these reasons, a piezo-actuator was used to provide stable position-control for planned, POI trajectories during the *RS*. In contrast with certain previous fast-scanning approaches in which a piezo-actuator was used to adjust the focal plane through employing a sinusoidal vibration of the objective for the purposes of sampling a populations of multiple neurons (Göbel et al., 2007), we chose to implement a unique single “sweep” trajectory for each neuron. This method was chosen because of the non-uniform distribution of POIs on the Z-axis due to unique and sporadic patterns of dendrite branching of each brain neuron's dendritic arbor. Our routine calculates the required trajectory to scan all POIs using a monotonically increasing path from *Z*_*min*_ to *Z*_*max*_ (Z-axis distance encapsulating all of the POIs) varying the position of the piezo-actuator to adjust the focal plane. The duration of the trajectory is based on the mechanical limitations (maximum velocity and acceleration) of the piezo-actuator and the required time the focal plane should exist at any particular position on the Z-axis to scan one or more POIs.

### AODs and High Speed POI Scanning

AODs are “solid-state” devices that can provide random-access control of laser beam positioning at high speeds and have negligible fly-back delay in comparison to scan mirrors. The “laser-scanning” mechanism of an AOD consists of passing a soundwave through a piezoelectric transducer coupled to a crystal (e.g., TeO_2_). Controlled changes in the piezoelectric modulate the crystal causing contractions and rarefactions in the substrate resulting in periodic changes in the refractive index similar to a diffraction grating, to refract the path of the laser. The deflection angle of the AODs, θ_*scan*_, can be modulated using the following relation,

$$\begin{array}{l} \theta_{scan}=\frac{\lambda f_{mod}}{v} \end{array}$$

where λ is the wavelength of the laser, and *f*_*mod*_, is the modulating frequency passing through the AOD crystal and *v* is the intrinsic, acoustic velocity of the material. The rate of change of the AOD is limited to the “access time,” *T*_*ACC*_, the time for the radio-frequency wave to propagate completely through the beam waist of the laser in the AOD deflecting the path of the laser to a different angle. *T*_*ACC*_ is defined as,

$$\begin{array}{l} T_{ACC}=\frac{\emptyset_{L}}{v} \end{array}$$

where ∅_*L*_ is the laser beam diameter.

### POI Scan

Each POI scan (Figure 3B) consists of a short line scan of 11 pixels, and 5 samples per pixel. The 5 measurement samples per pixel site are summed, and the combined intensity *I(k)* is then mapped to image space *I(x,y)*. This is done to account for the spatial drift and noise that is inherent in *in vivo* imaging experiments. The minimum time required to scan a POI is defined by the following sum,

$$\begin{array}{l} T_{POI}=\;T_{Buffer}+T_{ACC}{+\;T}_{SCAN} \end{array}$$

where *T*_*BUFFER*_ is the time required between samples and *T*_*SCAN*_ is the time for the AODs to scan from *f*_0_ to *f*_1_ shifting the path of the laser. The maximum allowable velocity of the piezo actuator, *V*_*Z*_*MAX*_, is constrained by *T*_*POI*_ where a required tolerance, *d*_*TOL*_, is defined to ensure the laser excites the coordinates of the POI to capture the fluorescent signal. *V*_*Z*_*MAX*_, is defined as follows,

$$\begin{array}{l} V_{Z\text{\_}MAX}=\frac{2d_{TOL}}{T_{POI}} \end{array}$$

### AOD POI Laser Deflection Programming

The relationship between image space coordinates and the AOD frequency input converts image POI coordinates to AOD instructions, which control the random-access, laser excitation position on the focal plane. Image space coordinates (i.e., locations of the POIs) are converted to AOD frequencies using the following,

$$\begin{array}{l} \left[\begin{array}{c} f_{0}\\ f_{1} \end{array}\right]=\left[\frac{f_{max}-f_{min}}{f_{BW}}\right]\left[\begin{array}{c} u_{0}-\frac{L_{POI}}{2}-0.5\\ u_{1}+\frac{L_{POI}}{2}+\;0.5 \end{array}\right]+\left[f_{min}\right] \end{array}$$

where each pair of frequency instructions 〈*f*_0_, *f*_1_〉, commands the AODs to execute a swept range from *f*_0_ to *f*_1_, between the minimum frequency of the AOD, *f*_*min*_ to the maximum frequency, *f*_*max*_. The swept range represents a short line scan and directs the laser through the POI on the arbor of the neuron to excite fluorophores and capture the resulting fluorescence light sample, *I(k)*. *u*_0_ is the start of the line scan and *u*_1_ is the end of the line scan in image space. *L*_*POI*_ is the length of the line scan, 11 pixels in image space. Since AODs are mounted orthogonally with-respect-to each other, duplicate coordinate pairs are sent to each other resulting in a diagonal sweep across the imaged points *u*_0_, *u*_1_, in the X-Y plane.

### Piezo-Actuator and AOD Synchronization

**Figure 4:**
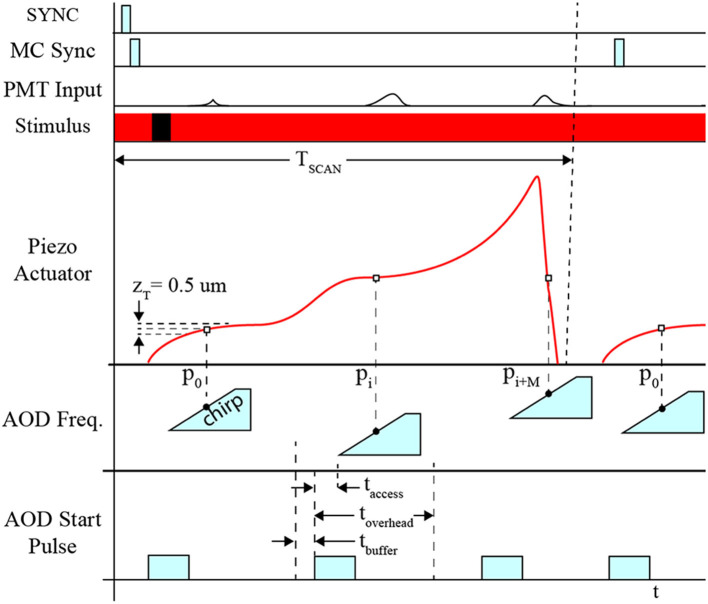
Timing diagram for system synchronization. Synchronization between devices is coordinated using the sample clock signal, T_CLK_. Initially, the start signal (*START*) is activated indicating the beginning of the measurement sequence. All PMT channels initiate recording and AOD “chirps,” which are responsible for deflecting the path of the laser for line scans for F3D or FS measurement sequences. The Z-stage command signal, *CMD-Z*, begins after *START*, and feedback is recorded providing an absolute comparison between the desired position and actual position measured.

The position of the piezo-actuator and deflection angle of the AOD are synchronized to coordinate the laser focal point on the neuron's dendritic arbor and cell body in 3D space to excite the fluorophores and detect the fluorescence emissions (Figure 4). This is achieved by acquiring a template sweep trajectory scan (prior to executing the *RS*) and scheduling each frequency pair instruction (i.e., X-Y-position of the laser on the focal plane) according to the time of the known position of the piezo-actuator (i.e., Z-position of the focal plane). The template scan is generated by executing the planned trajectory repeatedly over a 4 s interval providing a recorded output of the piezo-actuator position feedback. Using the position feedback, an AOD start-pulse signal schedule is generated for each frequency pair. Each start-pulse synchronizes the start of the frequency pair instruction in the AODs with the position of the piezo-actuator to ensure each 11-pixel line scan crosses through each POI.

### System Requirements

We experimentally determined the key temporal and spatial system requirements using image sampling of neural activity evoked by visual stimuli in the developing brain of the *Xenopus laevis* tadpole (Figure 1B). Baseline measurements were conducted on a conventional galvanomirror-based TPLSM (custom-designed, modified Olympus BX61, running Olympus Fluoview 1000).

### Temporal Sampling Considerations

Four temporal measurements were required in order to determine temporal scanning rates necessary to sample brain neuron synaptic and AP activity. These included:

1. $\bar{\tau_{Delay}}$; the average delay time between the beginning of the visual stimuli and the peak amplitude of the evoked intracellular calcium transient response,
2. $\bar{\tau_{R}}$ and $\bar{\tau_{F}}\;$; the mean time constants of the rising and falling times of the calcium transients;
3. $\bar{f_{\max}}$; the frequency bandwidth of the evoked, calcium signals.

**Figure 5:**
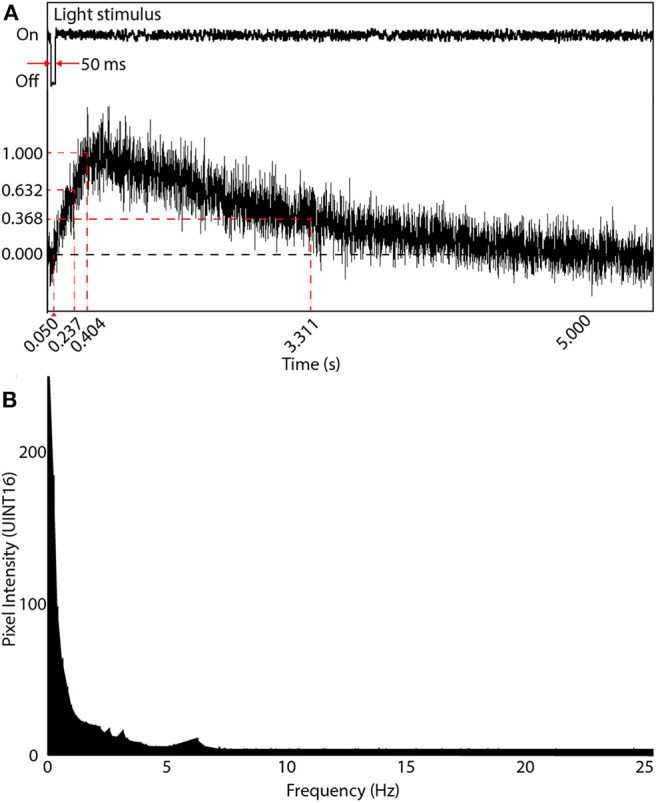
Visual-evoked calcium responses in a brain neuron using GCaMP6m. **(A)** A line scan of the neuron's soma was acquired at 1,000 Hz showing the evoked response following a 50 ms light OFF stimulus. The maximum intensity peak was 0.404 s after the OFF pulse. The rise time parameter (at intensity 1-1/e of peak) was measured 0.237 s after the OFF pulse, and the decay time parameter (intensity 1/e of peak) occurred 2.907 s after the peak of the signal rising transient. **(B)** Frequency response of **(A)** after applying the Fourier transform. Most information is in the very low frequency range (under 2 Hz), with diminishing power until 7 Hz, and noise thereafter.

When initially designing our random-access TPLSM system, we calculated the temporal sampling considerations based on the 6th generation GCaMPs that were at the time, the latest publicly available. We experimentally obtained the temporal data of the system from neurons expressing GCaMP6m in the optic tectum of head-mounted tadpoles. Calcium transients in dendrites and the soma were evoked by light stimuli produced by an LED. We subjected the tadpoles to repeated 50 ms OFF square pulses presented at 8–10 s intervals. Evoked responses were sampled at five locations on the neuron's dendritic arbor and at the soma. Averaged responses from 4 OFF events were used to determine the delay between stimulus onset and event peak ($\bar{\tau_{Delay}}$) as well as the parameters for the temporal rise to peak ($\bar{\tau_{R}}$) and fall to the baseline ($\bar{\tau_{F}}$). $\bar{\tau_{Delay}}$, $\bar{\tau_{R}}$ and $\bar{\tau_{F}}\;$were recorded experimentally as 0.404, 0.237, and 2.907 s, respectively (Figure 5A).

A Fast Fourier Transform (FFT) was then applied to each time-domain signal to acquire the frequency domain response. The average of all FFTs (Figure 5B) indicated most of the signals existed around 3 Hz. Thus the Nyquist sampling rate, *f*_*Nyq*_, during comprehensive imaging should be at least 6 Hz. Subsequent to the release of the 7th generation of GCaMPs we switched to using jGCaMP7s, which has both superior sensitivity and a slower decay rate then the 6th generation GCaMPs (dal Maschio et al., 2012; Dana et al., 2019). This allows for a potentially slower Nyquist sampling rate than previously calculated. When advanced spike deconvolution methods are required to achieve millisecond-precision on spike timings (Deneux et al., 2016), the faster planar scans are recommended due to the 200 Hz time resolution, and faster indicators such as jGCaMP7f can be used.

### Spatial Sampling Considerations

The requirement to capture POIs distributed across an entire neuron within the intact and awake brain requires imaging a large enough field-of-view to encompass the entire 3D dendritic arbor and the cell body over long periods. The minimum POI spatial resolution (*d*_*L*_) along the dendritic arbor is experiment-dependent and limited by scanning speed; however, should be <5 μm to identify neuronal subcompartments (Biess et al., 2011) and to saturate the domains of calcium transients allowing us to discriminate individual events. Thus, the optical resolution is required to be <2.5 μm to satisfy the Nyquist spatial sampling requirement.

### Temporal and Spatial Scanning Limitations

The temporal and spatial scanning limitations define the rates for the minimum scanning frequency, *f*_*Nyq*_, required for calcium imaging. This rate will vary based on the number of POIs, *N*_*POI*_, on each unique neuron arbor and is a function of the total dendritic branch length, *L*_*T*_ and the spatial resolution, *d*_*L*_. The minimum POI scanning frequency, *f*_*s*_, required can be calculated as follows,

$$\begin{array}{l} f_{s}\geq 2f_{Nyq}\\ f_{Nyq}={\left(N_{POI}\cdot T_{POI_{SCAN}}\right)}^{-1}\approx \;{\left(\frac{L_{T}}{d_{L}}\cdot T_{POI_{SCAN}}\right)}^{-1}\\ f_{s}\geq \;2{\left(\frac{L_{T}}{d_{L}}\cdot T_{POI_{SCAN}}\right)}^{-1} \end{array}$$

where *T*_*PO*_*I*__*SCAN*__ is the time required for the system to scan one POI.

### Microscope Driver Software Design

One of the overarching goals for our random-access TPLSM system design was to create a modular and flexible architecture. We believed that by partitioning the system's responsibilities and by maintaining a loosely coupled design, we would greatly simplify and expedite system modifications in response to future needs or technological innovations. For this reason, the system was designed using the Actor Framework (AF) concept (Hewitt et al., 1973), which supports implementing large, queued-message handler systems. The system was implemented using object-oriented design/programming for the AF [e.g., National Instruments LabVIEW (Kerry, 2012)], and allows for scalability and easier maintenance as the system expands over time. In general, the AF subdivides the system routines into “actors” or primitives that respond to messages from other actors and execute local routines with a minimal amount of overhead. By keeping routines fast, this avoids long synchronous processing, which enables asynchronous event-based processing that can require precise timings.

### Graphical User Interfaces

**Figure 6:**
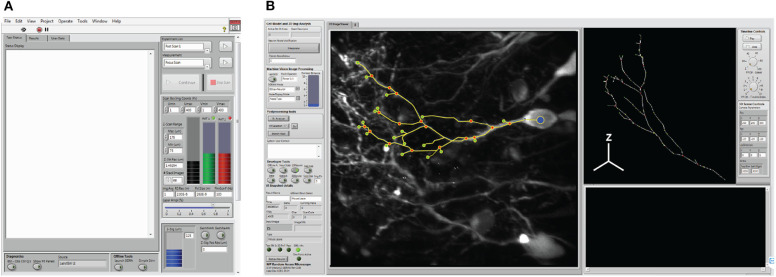
System user interfaces. **(A)** The measurement user interface (i.e., *Msmt. UI*) provides user access for hardware selection, and experimental design. **(B)** The cell trace and analysis toolkit user interface (*CTAK UI*) serves as an interface for drawing the initial 3D structure of the neuron, correcting minor positional errors following scans, and for post-analysis following either the *F3DS* or *FSS* routines.

Interactions between the operator and the system are facilitated through a measurement user interface (i.e., “*Msmt. UI”*, Figure 6A), and the Cell Trace and Analysis Kit user interface (i.e., “*CTAK UI”*, Figure 6B) to define experiments (i.e., individual or sequential sets of measurements) and draw the 3D neuron morphologies for experiments. These interfaces provide access to hardware parameters, and experiment design/post-processing tools respectively.

The *Msmt. UI* allows the operator to design scanning experiments, and set parameters required by the hardware peripherals. Experimental measurements (e.g., type of scan and number of repetitions of each type of scan) are arranged in order of execution, defined by the operator, and stored as an instance of the “*Experiment*” class. Each *Msmt*. in the *Experiment* executes the following sequence of overridden methods:

1. *Initialize*: preliminary settings for the measurement
2. *Configure*: requests resources to execute the measurement
3. *Acquire*: records the data from the input channel(s)
4. *Measure*: performs calculations using the raw data
5. *Close*: Releases resources held during the acquisition stage and signals completion.

The *CTAK UI* acquires operator input during experiments and provides a visual display of the results of each *Msmt*. During experiments, the operator can draw/modify the spatial organization of the POIs defining the neuron's dendritic arbor. This 3D structure is stored within an instance of the *Neuron* class and relates the spatial information, such as the locations of each POI, to the synaptic/AP patterns of activity recorded during comprehensive imaging (Figure 1D). The *Neuron* class is a descendant of the *Tree* class and stores POIs as a conventional tree structure comprised of a doubly linked list of *Branch Node* objects.

### Proposed Event Sequence for Comprehensive Imaging

**Figure 7:**
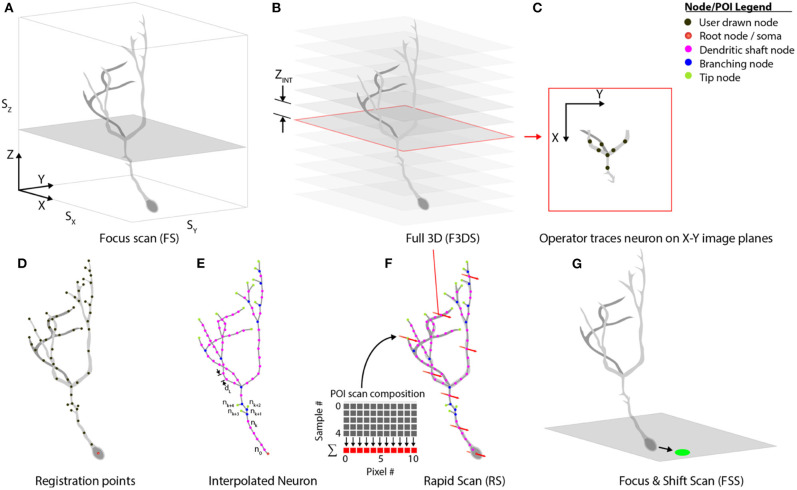
Scan class hierarchy and scan types. **(A)** The focus scan (*FS*) allows the operator to adjust the position of the neuron in 3D-space, and defines the boundaries during scanning. **(B)** The Full 3D scan (*F3DS*) images the volume defined in the *FS* at an interval along the Z-axis, *Z*_*INT*_, which is defined by the operator. **(C)** After the *F3DS* is acquired, the operator traces the neuron on each X-Y image planes forming a **(D)** skeletonized frame of the neuron in 3D space. **(E)** The registration points, drawn by the operator, are then linearly interpolated over the entire arbor using a spatial separation distance of *d*_*L*_. **(F)** The POIs are scheduled for scanning and the volume rapid scan (*V-RS*) is executed scanning all interpolated POIs at calcium imaging rates. **(G)** Following the RS, the user has an opportunity to make adjustments to the position of the neuron in 3D space to account for minor position shifts.

Our system separates comprehensive imaging of brain neurons into a sequence of pre-defined *Msmts*. executed by the system actors. These measurements are designed to be composable, extendable steps, and those available with the system are illustrated in Figure 7. An initialization routine begins by executing a focus-scan (*FS*, Figure 7A), a repeating X-Y scan allowing the operator to adjust the X, Y or Z-position of the specimen and set the imaging volume-boundaries encapsulating the neuron. These boundaries define the maximum limits to all scanning routines acquired during the experiment. A full 3D scan (*F3DS*, Figure 7B) is acquired and consists of a stack of *N*_*IMG*_ images with a resolution of S_X_ × S_Y_ pixels and separation distance, *Z*_*INT*_. This first *F3DS* provides the operator a representation of the neuron's 3D arbor on X-Y image planes. In Figure 7C, an illustration of one X-Y image in the stack shows the acquired cross-section of the neuron residing on one image plane.

The stack of X-Y image planes allow the operator to manually trace the neuron's full dendritic arbor and cell body in the *CTAK UI*, creating a skeletonized, 3D-frame (Figures 1C, 7D). The initial 3D-frame defines key registration points along each branch on the neuron. The initial drawing of the neuron typically requires approximately 15 min. Given the delay following the conclusion of the operator tracing the neuron, a second *F3DS* routine is executed to provide minor adjustments to the position of the neuron on X, Y, and Z-axes to account for sample drift. Further ancillary *F3DS's* are employed typically at 10 min intervals throughout the experiment to detect and accommodate growth or position drift. Following the second *F3DS*, the operator-drawn, skeletonized, neuron is linearly interpolated along the dendritic arbor at an operator-selected resolution (e.g., 2 μm spacing) in 3D-scanned space defining the location of the POIs intended for scanning (Figure 7E).

The rapid scan (*RS*, Figure 7F*)* is the comprehensive, 3D scan used to acquire the synaptic activity and AP firing information along the arbor and cell body of the neuron at all of the POIs on the neuron. The *RS* is initiated concurrently with the visual stimulation routine. During the visual stimulation routine, stimuli are presented to the eye contralateral to the optic tectum containing the neuron. While the stimuli are being presented to the eye, functional activity is acquired. At the end of the stimulation sequence, a focus-and-shift routine (*FSS*, Figure 7G) is executed to accommodate minor shifts with respect to the expected position of the cell body. A *F3DS* is then acquired and the operator can adjust either the location of individual POIs or the location of the entire neuron if required. This sequential set of measurements, (*RS, FSS*, and *F3DS)* and the visual stimulation routine continues as scheduled in *Experiment*.

### Rapid Scanning Scheme

To achieve comprehensive analysis of the entire neuron, the system requires a scanning routine to sample all POIs and minimize the period, **T**_**G**_, between POIs. Sufficiently fast sampling rates are required to detect the fast calcium transients mediated by synaptic activity throughout the expanse of the dendritic arbor and APs in the soma. Scanning routines must take into account that dendritic arbor morphologies are complex and the POIs along the arbor have a non-uniform spatial distribution along the Z-axis. Fully imaging a neuron's entire set of POIs requires rapidly repositioning the relatively heavy microscope objective from focal plane-to-focal plane, and coordinating the AOD laser-deflection angle with the position of the focal plane in order to capture all POI in each plane.

### System Validation

**Figure 8:**
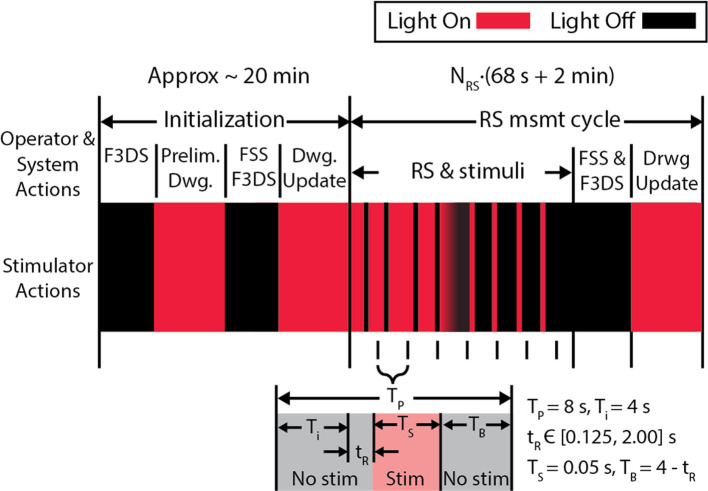
The experiment protocol used to validate the microscope. An initial stimulus, consisting of alternating screens “ON” and “OFF” are displayed during the initial *F3DS*, after which the user draws the initial morphology in the *CTAK UI*. The *RS* executes immediately after the initial drawing of the neuron structure, and stimuli are displayed while recording the 3D, evoked activity in the dendritic arbor AP firing. In this stimulation routine, 9 stimuli are presented in sequence-−4 OFF pulses on a bright background a transition shift from the bright background to the dark background and 4 ON pulses on a dark background. Each stimulus is displayed within an 8 s interval (*T*_*P*_) and has a buffer time, *T*_*B*_, before and after the stimulus.

Comprehensive imaging of sensory-evoked calcium activity in a visual stimulus processing neuron in the brain of the awake albino *Xenopus laevis* tadpole was used to validate this new microscope system. This model animal was selected due to its transparency, which allows direct imaging of brain neurons in awake and immobilized specimens. Moreover, the external development of tadpoles permits imaging of early vertebrate brain developmental events that typically occur in the womb in mammals (Sin et al., 2002; Ruthazer and Aizenman, 2010). Development of the tadpole visual system has been extensively studied (Engert et al., 2002; Sin et al., 2002; Dunfield and Haas, 2009; Li et al., 2011). The experimental routine in Figure 8, for system validation, was used to identify a tectal neuron's dendritic arbor morphology and observe the functional activity in response to a controlled, visual stimuli paradigm (Dunfield and Haas, 2009). The first epoch consists of 8 measurement cycles of the rapid-scan, *N*_*RS*_, with each cycle presenting 9 stimuli-−4 light-off pulses (OFF) on a bright background, a gradual transition shift from a bright background to a dark background and 4 light-on pulses (ON) on opposing background. A simplified variation of this routine removing the ON pulses and the gradual transition shift and instead having 9 OFF pulses on a bright background was subsequently created.

### *Xenopus laevis* Preparation

Freely swimming albino *Xenopus laevis* tadpoles were reared and maintained in 10% Steinberg's solution (Dunfield and Haas, 2009). In order to transfect brain neurons for expression of calcium sensors we used single-cell electroporation of plasmid DNA (Haas et al., 2001). Seven days post-fertilization, tadpoles were anesthetized using 0.01% solution of MS-222 (A5040-25G, Sigma-Aldrich). A borosilicate micropipette (BF150-75-10, Sutter Inc.) pulled on a micropipette puller (P-97, Sutter Inc.) was backfilled with a solution containing 3 μg/μL plasmid DNA encoding the a green calcium-sensitive fluorophore, jGCaMP7s and a red space-filling fluorophore mCyRFP in calcium-free, ringers solution (in mM: 116 NaCl, 1.2 KCl, 2.7 NaHCO_3_). The filled micropipette was then inserted into the optic tectum of the tadpole and an Axon 800A Electroporator delivered a train of voltage pulses to induce electroporative transfection. Prior to experimentation, tadpoles were paralyzed in a bath using 2 mM panacronium dibromide (0693/50, Tocris). All experimental procedures were conducted on Stage 49 tadpoles (Nieuwkoop and Faber, 1994) according to the guidelines of the CCAC and were approved by the Animal Care Committee of the University of British Columbia's Faculty of Medicine.

### Animal Stimulation Chamber With Visual Stimulator

**Figure 9:**
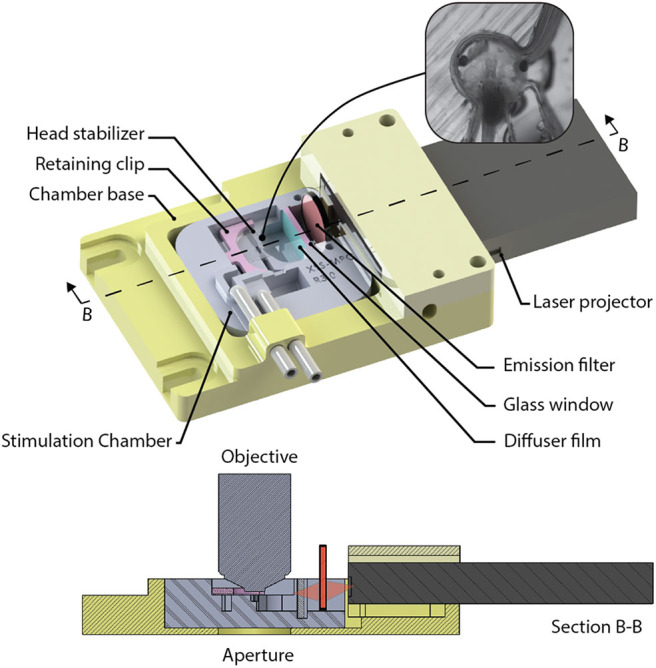
The visual stimulation chamber was designed to allow simultaneous imaging and visual stimulation by projecting red and black images to screen (diffuser film) using a laser projector (inset). The head of a tadpole is stabilized inside a small cavity that has an opening allowing the tadpoles eye to view the screen.

A custom-designed imaging chamber (Figure 9) is used to stabilize the head of the tadpole while imaging, to provide the specimen with oxygenated solution, and to provide visual stimuli to the contralateral eye. Stabilization of the animal is required during imaging routines to prevent positional drift by minimizing tadpole movement using a formfitting chamber. The design of the chamber was based on tadpole morphology and tested using *in vivo* time-lapse imaging of neuronal structures. Physical dimensions of Stage 49 *Xenopus laevis* were used to determine the spatial requirements. Tadpoles with on overall body length of 11.0 ± 0.5 mm have head diameters of 3.3 ± 0.2 mm, and eyes are offset by 17.4 ± 2.5 degrees from the transverse axis. *Xenopus laevis* brain neurons fit within a cubic volume of 100 μm^3^. Thus, we set the minimum FOV (*S*_*X*_ and *S*_*Y*_), and the axial requirements, *S*_*z*_ to ensure that the neurons will fit into a volume with sides of at least 100 μm. The stimulation chamber was created by printing a negative mold using polyactide (PLA) using a 3D-printer (Creatr Dual Extruder, Leapfrog), and filling the mold with polydimethylsiloxane (PDMS, Sylgard 184, Dow Corning). The head of the *Xenopus laevis* was stabilized in the chamber using a 0.2 mm thick, square sheet of cellulose acetate secured by a 3D-printed, PLA, C-clip. The *Xenopus laevis*'s eye was coaxial with the normal of the visual stimulator screen. The resulting field of view of the screen available to the eye is 78° on the horizontal and 44° on the vertical plane, while being submersed with several millimeters of physiological solution. Clearance between the screen and the optical axis of the microscope allows a water immersion objective with a diameter ≤ 33 mm, with shank angle greater or equal to 26° from the aperture of the objective. Objectives this size can accommodate a maximum of 9 mm motion of the objective in X, or Y directions controlled via three manual stages (X_M_, Y_M_, and Z_M_) before obstruction with the chamber or screen. A mini laser-projector (ShowWX+/PicoP, Microvision), mounted directly to the stimulation chamber, projects the stimulus image on a diffuser film (Inventables, 23114-08) that is mounted on a section of a glass slide (VWR, 16004-386) with PDMS, which is the interface between the physiological solution and the project image.

## Results

### Validating the Optical Train

The optical train was evaluated with measurements acquiring the amount of power reaching the back aperture, the axial and lateral optical resolution, and the field-of-view of the microscope. The power of the laser was measured before and after the prism used for spatial compensation and at back aperture of the objective. The power being emitted from the Ti-Sapphire, using maximum dispersion compensation was 1.94 W out of the Ti-Sapphire laser, 1.35 W after the beam expansion, and 0.35 W maximum at the back aperture of the objective using a wavelength of 910 nm to maximize the excitation of jGCaMP7s.

The axial and lateral resolution of the microscope, using two-photon excitation at 910 nm was measured using 0.1 μm diameter green fluorospheres (F-8803, Thermo Fisher Scientific). The lateral resolution was measured to be an average of 0.34 ± 0.3 μm full-width at half maximum (FWHM, N = 9) by 1.24 ± 0.3 μm FWHM (*N* = 9), which were the major and minor axes over the entire FOV of the image. The difference accounts for the spatial dispersion caused by the AODs due to the varied frequencies over the scan. This value remains constant between *RS* and *F3DS* since the same frequency dwell time is used. The axial resolution was measured to be 2.5 ± 0.7 μm.

Tracking the edges of a fluorescent standard (1951 USAF Target #57-855, Edmund Optics, Group 4—Element 6), determined a maximum FOV using the AODs and the 60X objective to be 112 × 112 μm. Using the same standard, the FOV of the digital camera was measured to be 288 × 216 μm at a resolution of 2,560 × 1,920 pixels.

### Piezo-Actuator Validation

**Figure 10:**
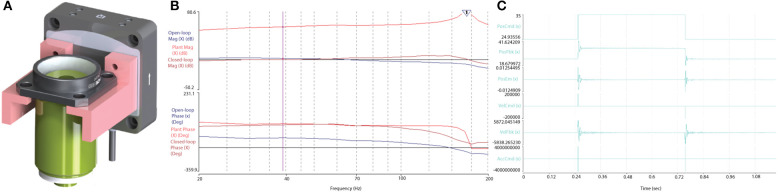
Optical train and piezoactuator performance validation. **(A)** The 0.176 Kg objective assembly mounted on the Aerotech, piezo actuator, and **(B)** the Bode plot output following a stability test proved the actuator stable up to ~125 Hz. **(C)** The Z-stage, piezo actuator's response to a step command over 100 and 10 μm, respectively. The Piezo achieved a maximum velocity of 0.043 m/s with a following error less than ± 5 × 10^−6^ m, and a settling time of <5.3 × 10^−3^ s.

The performance of the piezo-actuator was evaluated for its dynamic range and response to a step-input to determine the velocity, acceleration and settling response to define the limitations during the *RS* and *F3DS* scans. The dynamic range of the Z-actuator and 0.176 Kg payload (objective and mount, Figure 10A) was measured using the vendor's onboard parameter tuning application for piezo actuators (Aerotech EasyTune™) and found to be stable up to approximately 125 Hz (Figure 10B). The maximum velocity, *V*_*Z*_*MAX*_, of the Z-stage was experimentally determined by observing the output to a step-response by observing the settling time, *T*_*S*_, with an error <0.5 μm. The piezoactuator was subjected to a step response of 100 μm (Figure 10C). Under the loaded conditions, a maximum velocity of 0.043 m/s was obtained in both directions with a settling time of <2.1 × 10^−3^ s. Acceleration values of <60,000 m/s/s were found to provide stability ensuring following errors of <0.5 × 10^−6^ m.

### Rapid-Scan Position Validation

The *RS* was validated to ensure that the X-Y position of the laser was synchronized with the position of the focal plane ensuring that the laser excited the 3D-position in space defined by the coordinates of the POI. Fluorospheres, 0.1 μm in diameter (F-8803, Thermo Fisher Scientific), were embedded and distributed within a 300 μm thick layer of Sylgard 184 providing sufficiently spaced, and immobile targets for analysis. A *F3DS* scan captured a volume of 112 × 112 × 125 μm (slightly greater than the volume of neurons sampled for analysis) and a total of 10 beads were drawn in the *CTAK UI*. The *RS* scan was executed and the Z-actuator provided position feedback from its capacitive sensor with an average relative error of ± 3.9% Δ*F*/*F*_0_ (*N* = 10 beads). The capacitive feedback from the Z-actuator was compared with the desired position of the POIs and an error of 0.375 × 10^−6^ ± 0.125 × 10^−6^ m (*N* = 8 motion trials of the same trajectory, 68 s per motion trial) was measured with respect to the entire motion profile with an error of 0.375 × 10^−6^ ± 0.125 × 10^−6^ m (*N* = 1270 measurements) on the Z-axis for all nodes.

### Experimental Validation and 4D Data Collection

**Figure 11:**
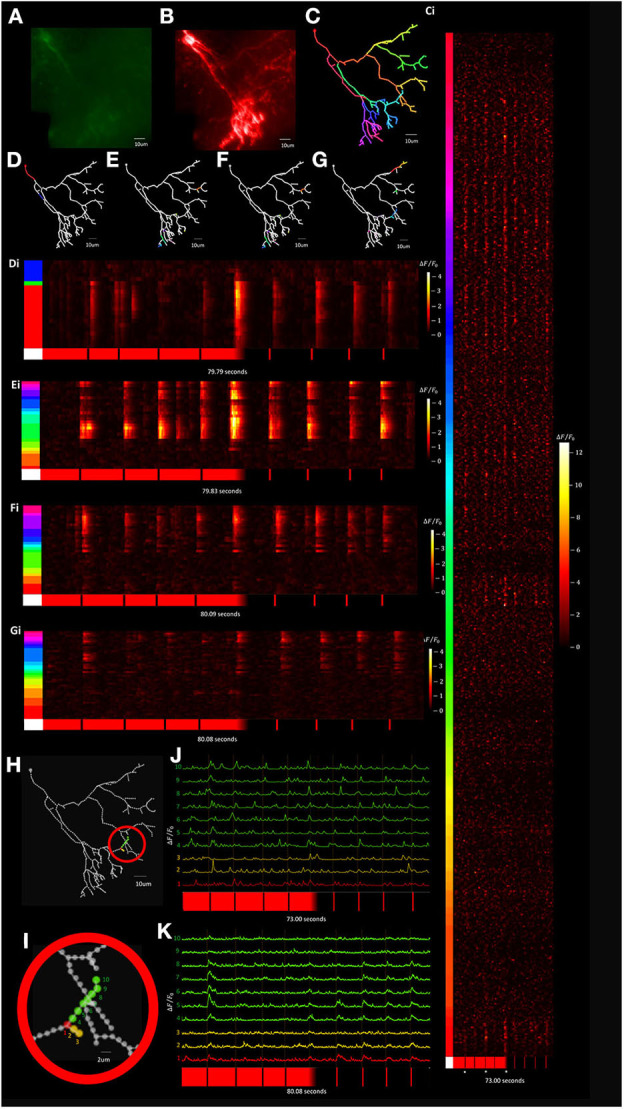
Visually evoked tectal neural activity can be recorded across the complete dendritic arbor and soma using random access TPLSM. **(A,B)** A standard deviation projection image in the green **(A)** and red channels **(B)** of a *Xenopus* tectal neuron expressing jGCaMP7s and mCyRFP created from a *F3DS* stack of images is shown. **(C)** The user-drawn POIs tracing out the structure of the neuron. A total of 609 POIs are drawn located at 2 μm distances from their nearest neighbor. **(Ci)** Δ*F*/*F*_0_ output from the resulting *F3DS* of the 609 POIs, collected at 3 Hz over a 73 second interval. Responses can be seen for four 50 ms “OFF” stimuli, a “transition shift” stimulus and four 50 ms “ON” stimuli at spaced at pseudorandom time points. White asterisks indicate evoked somatic events. **(D–G)** The user-drawn POIs tracing out the structure of the neuron with the colored POIs sampled using a series of planar *RS* **(Di-Gi)** collected between 204 and 246 Hz with a similar experimental protocol as used previously (C-Ci). **(H–I)** A series of POIs that were recorded in both a 3 Hz *F3DS* scan and 232 Hz planar *RS*. **(J)** The traces for the POIs recorded at 3 Hz over 73.00 s. **(K)** The traces for the POIs recorded at 232 Hz over 80.08 s.

Visually evoked calcium activity was recorded in individual *Xenopus* tectal neurons expressing jGCaMP7s and mCyRFP1 through single-cell electroporation and used to demonstrate the system's capabilities of the routine. We initially validated the platform's capability to record stimulus-evoked calcium events with a temporal resolution necessary to be accurately registered as evoked events by automated detectors in *Xenopus* tectal neurons expressing GCaMP6m (Sakaki et al., 2018). However, upon release of the 7th generation of GCaMPs we switched to jGCaMP7s due to its superior sensitivity and longer decay time which improves the platform's capability of detecting short duration, local calcium transients across a large number of points in a 3D space, such as is the case with synaptic inputs (Dana et al., 2019). Both the higher Δ*F*/*F*_0_ and slower decay rate of jGCaMP7s maximize the number of samples collected above noise, giving high confidence that transients are detected in response to a stimulus. A typical result set, shown in Figure 11, shows a standard deviation projection of a neuron (Figures 11A,B) for images collected during the *F3DS*. The user-drawn, computer interpolated 3D-structure is shown in Figure 11C. Calcium data for each of the 609 POI collected at a rate of 3 Hz is shown for the initial time period in Figure 11Ci and shows Δ*F*/*F*_0_ traces for each of the POI recorded representing each raw “line-scan” for each POI on the neuron. As indicated immediately beneath each of the line-scans four 50 ms OFF stimuli are presented at pseudorandom time points on a bright background, the background is gradually shifted from bright to dark over several seconds and then four 50 ms ON stimulus are presented at pseudorandom time points. An in-house designed automated event detection system (Sakaki et al., 2018) was employed to detect the number of stimulus that evoked action potentials in this neuron (2/4 OFF stimuli, 1/1 transition shift stimulus and 0/4 ON stimuli). Four separate planar *RS* (i.e., no moving Z-stage, scanning is exclusively provided by the X-axis and Y-axis AODs) were performed on this same neuron using a similar experimental protocol as was used in the *F3DS* scan (Figures 11D–G,Di–Gi). While the planar *RS* lack the comprehensive imaging capability of the *F3DS* scan, they provide greater temporal resolution, recorded at scanning rates between 204 and 246 Hz, each recording POIs on a single Z-plane cutting through the neuron. A comparison of the individual traces for a section of the neuron sampled both with a 3 Hz *F3DS* scan (Figures 11H,J) and a 232 Hz planar *RS* (Figures 11I,K) demonstrate the advantage of an increased sampling rate for recording stimulus-evoked calcium microdomains that are potentially synaptic.

**Figure 12:**
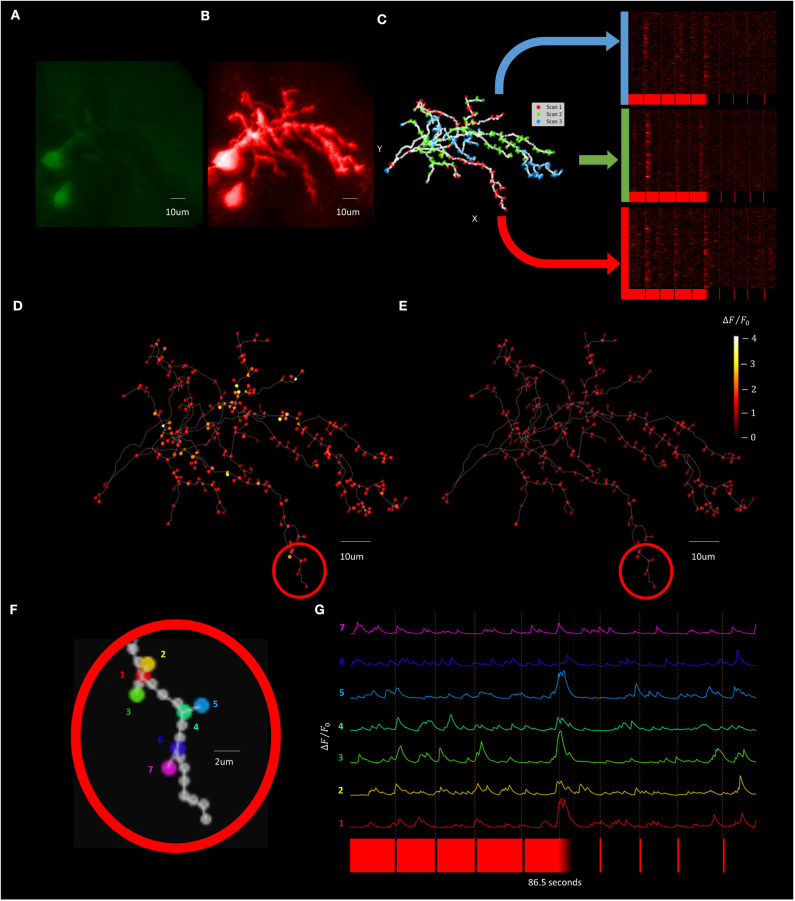
Visually evoked synaptic calcium transients across the neuron can be recorded with increased temporal resolution using Segmented Scanning. **(A,B)** A standard deviation projection image in the green **(A)** and red channels **(B)** of a *Xenopus* tectal neuron expressing jGCaMP7s and mCyRFP created from a *F3DS* stack of images is shown. **(C)** The user-drawn POIs tracing out the structure of the neuron with the POIs that are sampled in each sub-scan of the Segmented scan are colored and the subsequent visually evoked calcium activity is shown. A total of 324 POIs located at the soma, branch points, filopodia bases and tips are collected at a rate of 6 Hz. **(D,E)** The maximum of the average Δ*F*/*F*_0_ evoked responses for both a series of four pseudorandom 50 ms “OFF” **(D)** and four 50 ms “ON” stimuli **(E)**. **(F)** A series of POIs recorded corresponding to filopodia tips and bases and **(G)** the individual calcium traces of those POIs.

To improve temporal resolution relative to the *F3DS* scanning method while still maintaining a dataset containing recordings from across the dendritic arbor a “Segmented Scanning” was developed. In this method, the 3D structure of the neuron (Figures 12A,B) is divided into 3 compartments (Figure 12C). Furthermore, the majority of interstitial nodes are excluded from scanning, with sampling only occurring at the soma, branch points, filopodia bases and filopodia tips (a total number of 324 POIs for this neuron), where previous studies demonstrate there is an enrichment in the density of synapses in these neurons (Li et al., 2011). Fluorescence data from each third is serially collected in three imaging epochs and the data from all three scans is then combined to produce a reconstructed recording of both OFF-stimulus evoked (Figure 12D) and ON stimulus evoked calcium transients (Figure 12E). Figures 12F,G shows a the Δ*F*/*F*_0_ traces of series of POIs at the tips and bases of filopodia recorded using a Segmented Scan at a rate of 6 Hz (4 filopodia out of a total of 174 on the neuron), where spatially restricted (2 μm) stimulus-driven calcium currents are present at the tips of filopodia that represent synaptic activity.

**Figure 13:**
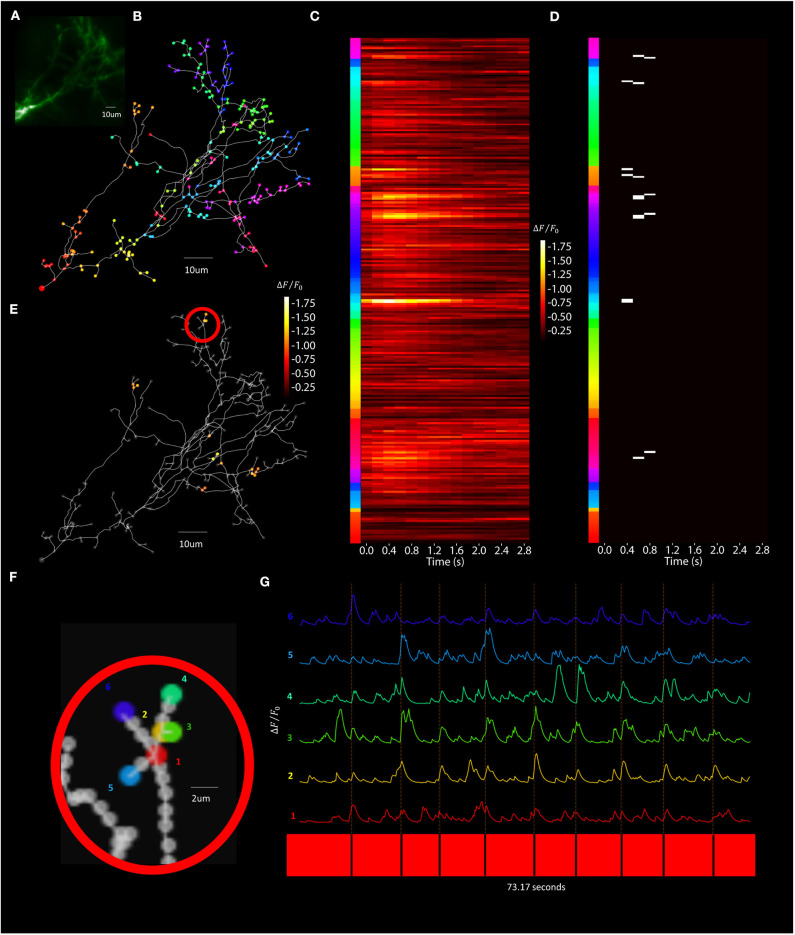
Visually evoked synaptic-localized glutamate transients across the neuron can be detected using Segmented Scanning. **(A)** A standard deviation projection image in the green channel of a *Xenopus* tectal neuron expressing shown sfGFP-iGluSnFR-A184S. **(B)** Locations of a total of 259 POIs sampled across the soma, dendritic branch points, filopodia bases and tips. **(C)** Stimulus-evoked iGluSnFr Δ*F*/*F*_0_ response for 3 s post-stimulus at each POI, collected at a rate of 6 Hz and averaged over 9 50 ms “OFF” stimuli. **(D)** Transient events detected using a matched filter algorithm (Sakaki et al., 2018), identifying evoked increases that match Super-folder-GFP-iGluSnFR-A184S dynamics ($\bar{\tau_{R}}$ = 5 ms, $\bar{\tau_{F}}$ = 150 ms) and a window size of 8. **(E)** Locations and strengths of the responses located spatially across the arbor. **(F)** A series of POIs recorded corresponding to filopodia tips and bases and **(G)** the individual iGluSnFr traces of those POIs.

The ability of this platform to record stimulus-evoked neuronal glutamate release onto a neuron was also tested using *Xenopus* tectal neurons expressing the non-ratiometric fluorescence based glutamate sensor Super-folder-GFP-iGluSnFR-A184S (Marvin et al., 2013, 2018) through employing single-cell electroporation, with a result set shown in Figure 13. Segmented Scanning using 9 OFF stimuli was performed on an individual neuron expressing the construct (Figure 13A) and a total of 259 POIs were collected at a rate of 6 Hz. Active areas of glutamate release could be analyzed by calculating the stimulus-evoked response for each POI, using the average Δ*F*/*F*_0_ change between 0 and 3 s after the 9 stimuli (Figure 13C). Responses were identified using a matched filter algorithm (Sakaki et al., 2018), to the expected iGluSnFR dynamics ($\bar{\tau_{R}}$ = 5 ms, $\bar{\tau_{F}}$ = 150 ms) using a window size of 8 samples (Figure 13D) and localized back to their position in the arbor (Figure 13E). Figures 13F,G show the traces of a subset of 6 POI on 4 filopodia demonstrating recordings of spatially restricted, stimulus driven glutamate release onto filopodia tips.

## Discussion

Our AOD TPLSM achieved comprehensive imaging of brain neurons while providing an open-source blueprint for the full software and hardware systems. The system successfully detected activity at subcellular resolution on regions of the neuron's dendritic arbor and cell body. We demonstrated that that our system can capture calcium-based, fluorescence activity at 2 μm resolution at 3 Hz over an entire neuron's dendritic arbor (Figure 11). Through employing Segmented Scanning (Figures 12, 13) this system is able to increase the sampling rate to 6 Hz while generating trace data from across all filopodia and branchpoints across the dendritic arbor. Furthermore, through the planar *RS* this TPLSM can provide higher temporal resolution sampling (i.e., >100 Hz) in continuous sections of the neuron within single focal planes (Figure 11). In conjunction, these multiple scanning modes accord users of this platform a substantial degree of flexibility in tailoring its function to their experimental design.

One of the major goals of this work was to provide a software, mechanical and electrical framework to serve as a template for comprehensive imaging, as well as for future TPLSM development. We achieved this by partitioning this system into modules with distinct areas of responsibility. The low dependency between modules allowed tasks to run independently from each other (e.g., measurement tasks such as the *RS*) while system maintenance/monitoring was consecutively handled. It is evident that the overhead throughout the development of the system using LVOOP/Actor Framework was initially high. However, the benefit of the system's versatility with respect to the scalability of the platform (e.g., other measurements, additional hardware configurations, other modes of imaging) greatly overshadows the burden of the initial, implementation investment.

Our UI designs (*Msmt UI* and the *CTAK UI*) handled the system/experiment administration and seamlessly integrated the operator's user-in-the-loop tasks into the experiment (e.g., drawing the neuron, updating POI positions). Up-to-date, *F3DS* and *FSS* information from the scanned neuron provided the user with the means to identify and maintain the relationship between the neuron's morphology and the TPLSM system analyzing each POI extracted from the morphology. It is apparent that having a user-in-the-loop will have obvious advantages, depending on the skill of the operator (e.g., quickly identifying small cell features, tracing the neuron), and disadvantages depending on the duration of the experiment (e.g., operator fatigue and fatigue-related mistakes). However, given the current limitations of machine vision algorithms' accuracy in localizing the topography of the neuron's arborization (Peng et al., 2011), we hope to develop or encourage the development of fully automated methods of identifying and maintaining the neuron's structure and eliminate the need for the user-in-the-loop.

Our *RS* utilizes a hybrid AOD-piezoactuator combination to acquire calcium-related activity from POI scans in 3D by acquiring all of the POIs using a planned trajectory. The planned trajectory synchronizes the motion of the piezoactuator with the laser deflection angle of the AODs on the X-Y plane. Using this method, we have achieved comprehensive imaging rates up to 4 Hz and have sustained this method over durations of 68 s with measured errors of no more than 0.375 × 10^−6^ ± 0.125 × 10^−6^ m throughout the duration of the experimental trials.

We acknowledge that an obvious bottleneck exists at the piezoactuator as a result of the mechanical motion. However, we believe we achieved the overarching goal of developing a versatile system for comprehensive imaging, which can be optimized through further design improvements and minimal effort, as current research has demonstrated that incorporating compact remote focusing technologies with acousto-optics has become feasible (Nadella et al., 2016). Furthermore, the simplicity of the design requires only basic skills for implementation to achieve initial imaging as opposed to more complex systems where remote focus designs are used or where optical trains are designed and dedicated to compensating for the dispersion of AODs.

In this work, we described an open-source, rapid-access, two-photon laser-scanning system for comprehensive sampling and analysis of neuronal activity. We demonstrated a system capable of extracting and creating a structural representation of a neuron and converting that structure into points-of-interest (POIs) to saturate sampling of activity in the cell body and throughout the entire dendritic arbor. Our system, using the list of POIs, creates a schedule for rapid-scanning, and coordinates acousto-optics and a high-speed, piezo linear-actuator to position a laser on each POI for rapid-scanning. We devised a 3D-printed chamber, and visual stimulator system to provide controlled sensory stimuli to awake immobilized animals. In conjunction, this enables us to record visually-evoked responses across the complete dendritic arbor and soma of an individual neuron with in an intact, awake developing brain. This is a novel capability and has broader appeal for the developmental neuroscience community, in particular allowing for the investigation of experience-driven neuronal growth and development of tuning.

Our versatile, open-source object-oriented, system software architecture was built using Actor Framework and LabVIEW “G” architecture using a powerful, established and commercially maintained API. This system architecture can be easily adapted for further experimental design as well as easily adapting the routines for other architectures, lending itself for future rapid prototyping as well as more mature designs in either research or for industrial purposes.

Our validated AOD-based random-access TPLSM with software is specifically designed to capture calcium biosensor fluorescence of neural activity throughout a neuron's entire dendritic arbor and soma in the intact and awake brain. Together, this hardware and software platform is capable of comprehensive imaging, required for understanding synaptic integration and neural encoding.

Additional documentation for this platform and instructions for further access to relevant resources is located at the UBC Dynamic Brain Circuits in Health and Disease GitHub page: https://github.com/ubcbraincircuits.

## Data Availability Statement

The datasets generated for this study are available on request to the corresponding author.

## Ethics Statement

The animal study was reviewed and approved by UBC Animal Care Committee and were in accordance with the Canadian Council on Animal Care (CCAC) guidelines.

## Author Contributions

KS redesigned and fully tested the AOD-based random access microscope and accompanying software for driving microscope and analysis and wrote the first draft of manuscript. KP produced initial design of the AOD-based random-access microscope and driver software. TD designed and tested the plasmid constructs optimized for this platform and performed the system validation experiments. PC developed segmented scanning, software for the microscope, and performed imaging analysis of the resulting data. KH supervised all design, construction and implementation efforts, and rewrote the manuscript.

## Conflict of Interest

The authors declare that the research was conducted in the absence of any commercial or financial relationships that could be construed as a potential conflict of interest.
